# Anti-atopic dermatitis effect of fish collagen on house dust mite-induced mice and HaCaT keratinocytes

**DOI:** 10.1038/s41598-023-41831-w

**Published:** 2023-09-09

**Authors:** Hye-Min Kim, Bo-Ram Jin, Jin-Sil Lee, Eun Heui Jo, Min Cheol Park, Hyo-Jin An

**Affiliations:** 1 Department of Herbology, College of Korean Medicine, Sangji University, Wonju, Gangwon-do 26339 Republic of Korea; 2 Department of Oriental Pharmaceutical Science, College of Pharmacy, Kyung Hee University, Seoul, 02447 Republic of Korea; 3 Department of Integrated Drug Development and Natural Products, Graduate School, Kyung Hee University, Seoul, 02447 Republic of Korea; 4 R&D Institute, Haewon Biotech, Inc., Bucheon, Gyeonggi-do Republic of Korea; 5 Department of Acupuncture and Moxibustion, Wonkwang University Korean Medicine Hospital and Research Center of Traditional Korean Medicine, Wonkwang University, Deokjin-gu, Jeonju, Jeollabuk-do Republic of Korea; 6 Department of Korean Medicine Ophthalmology and Otolaryngology and Dermatology, Wonkwang University Korean Medicine Hospital and Research Center of Traditional Korean Medicine, Wonkwang University, 460 Iksan-daero, Iksan, Jeollabuk-do 54538 Republic of Korea

**Keywords:** Drug discovery, Molecular biology, Diseases

## Abstract

Collagen, a major structural protein in mammalian tissues, is effective against skin wounds and osteoarthritis. Although bovine and porcine collagens have mainly been used, several potential risks of mammalian collagen have led to the use of fish collagen (FC) as an alternative. FC and its peptides are used as common cosmeceutical products because of their antihypertensive, anti-bacterial, and antioxidant activities. Despite the effects of FC on wrinkle reduction, UV-protection, and wound healing, the relationship between FC and atopic dermatitis (AD) has not yet been reported. Therefore, we investigated the anti-AD effects of FC against house dust mite (*Dermatophagoides farinae*, HDM)-induced AD in NC/Nga mice and TNF-α/IFN-γ-stimulated HaCaT keratinocytes. FC alleviated AD apparent symptoms, such as dermatitis score, transepidermal water loss, epidermal thickness, and mast cell infiltration upon declining pro-inflammatory cytokines and mediators, IL-6, IL-5, IL-13, TSLP, and TNF-α. The skin barrier protein, filaggrin, was also recovered by FC administration in vivo and in vitro. Immune response and skin barrier dysfunction are both mitigated by three routes of FC administration: oral, topical, and both routes via the regulation of IκB, MAPKs, and STATs pathways. In summary, FC could be a potential therapeutic agent for AD by regulating immune balance and skin barrier function.

## Introduction

Atopic dermatitis (AD) is a recurrently pruritic and chronic inflammatory skin disorder, placing the quality of patients' lives, roughly 3% of infants, 10–20% of children, and 3–10% of adults^1^. Cutaneous manifestations and location appear differently depending on age, with clinical hallmarks of AD, such as unbearable itching, erythema, xeroderma, abrasions, and lichenification^2^. The above AD symptoms are obscure to treat in that various etiologies work in combination rather than being limited to a specific one. Genetic and environmental factors are tied to the onset of AD, but among them, skin immunologic abnormalities and skin barrier dysfunction are the most representative^3^. Abnormal cytokines secretion from T-helper (Th) 1 and Th2 cells not only activates other inflammatory cells including eosinophils and mast cells, but also accelerates the breakdown of the skin barrier. And vice versa, impaired epidermal barrier function promotes allergen susceptibility and immune responses, which in turn, this vicious cycle exacerbates AD symptoms^4^.

At present, the general AD remedies are pharmaceutical emollients, topical corticosteroids, topical calcineurin inhibitors, and even systemic immunosuppressant drugs used in the case of severe symptoms for AD management^5^. Repetitive and long-term application of these drugs due to temporary anesis effects can lead to abuse and diverse adverse effects, such as skin atrophy, telangiectasia, steroid rosacea, perioral dermatitis, and acneiform eruptions, in severe cases, metabolic abnormalities, and bone development disorders in childhood^6^. For that reason, many scholars have been diving into excavating alternative and novel therapeutic approaches for AD treatment from natural resources^7^. As a natural resource, we focused on confirming the anti-atopic potential of collagen, especially fish collagen.

Collagen abundantly contained in the dermis is known to play an important role in the skin structural coherence, connected with the inhibition of wrinkle formation^8^. Existing experimental results have revealed the effect of oral and topical collagen supplements on skin disorders, and more potential and products are emerging in the cosmeceutical market^9^. Hydrolyzed collagen recovered the skin barrier function in mild AD and xerosis patients. Collagen peptides also attenuated inflammatory ailment by modulating Th2 cell activation via STAT1 pathway^10,11^. Further, oral intake of collagen peptide enhanced skin hydration and collagen density in UVB irradiation-induced skin model, clinical winkles patients, ex vivo, and hairless mice models^12,13^. Likewise, with previous studies indicating that fish collagen (FC) has anti-inflammatory, antioxidant, and anti-bacterial efficacy, FC exerts photoaging effects in melanocytes, skin fibroblasts, and hairless mice models^14^. Recently, studies on skin regeneration techniques through the combination of FC and other components as cross-linking agents have been actively carried out in vitro and in vivo^8,10^. Based on the aforementioned strengths of FC, we investigated anti-AD effect of FC in vivo and in vitro, as well as which routes of medication administration could synergistically relieve AD in HDM-treated NC/Nga mice.

## Methods

### Chemicals and reagents

For this study, dimethyl sulfoxide (DMSO), dexamethasone (DEX) (lot #: BCCC1349), and 3-(4,5-dimethylthiazol-2-yl)-2,5-diphenyl tetrazolium bromide (MTT) reagent were purchased from Sigma-Aldrich (St. Louis, MO, USA). Dulbecco’s modified Eagle medium (DMEM), fetal bovine serum (FBS), penicillin, and streptomycin were purchased from Life Technologies Corporation (Grand Island, NY, USA). ELISA kit for IL-6 was obtained from Abcam (ab100713, CB, UK). PowerUp™ SYBR^®^ Green Master Mix was obtained from Applied Biosystems (Foster City, CA, USA). The IL-5, IL-13, TSLP, tumor necrosis factor (TNF)-α, and β-actin oligonucleotide primers were purchased from Bioneer Corporation (Daejeon, Chungbuk, Korea). Primary antibodies for ERK (cat. no. 9102), p-JNK (cat. no. 9251), JNK (cat. no. 9252), p-p38 (cat. no. 9211), p38 (cat. no. 9212), p-TAK1 (cat. no. 4508), p-JAK2 (cat. no. 3776), and p-STAT3 (cat. no. 9145) were obtained from Cell Signaling Technology, Inc. (Danvers, MA, USA). The primary antibodies against p-IκB (cat. no. sc-8404), IκB (cat. no. sc-203), p-ERK (cat. no. sc-7383), p-STAT1(cat. no. sc-136229), STAT1(cat. no. sc-464), JAK2 (cat. no. sc-390539), STAT3 (cat. no. sc-482), and filaggrin (cat. no. sc-66192), β-actin (cat. no. sc-81178), and peroxidase-conjugated secondary antibodies were purchased from Santa Cruz Biotechnology, Inc. (Santa Cruz, CA, USA). Horseradish peroxidase-conjugated secondary antibodies were purchased from Jackson ImmunoResearch laboratories, Inc. (West Grove, PA, USA).

### Sample preparation

In this study, we purchased fish collagen from GELTECH (Gangseogu, Busan, Korea). Fish Collagen is manufactured by hydrolyzing fish gelatin extracted from the scales of *Tilapia mossambica* (Oreochromis genus). The product had a protein content of over 90%, a viscosity of 15 mps or lower at 40 °C, a pH range of 5.0–6.5 for a 15% solution, and a moisture content below 10%. This collagen purification was completed through hydrolysis followed by filtration, sterilization, parching, and sifting, as modified from a patented manufacturing process of collagen peptide (KR100647033B1).

### Animal experiment

**Figure 1:**
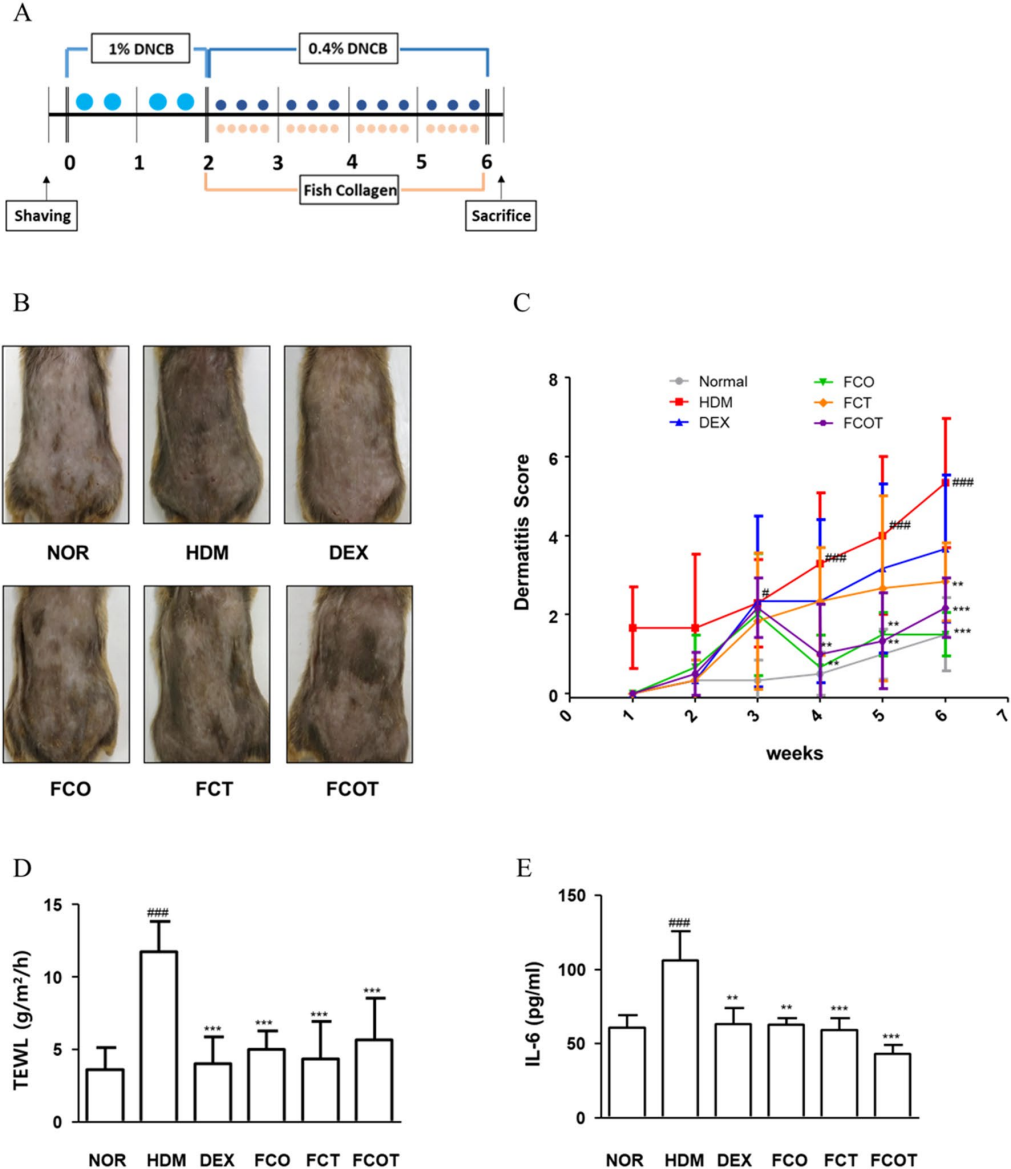
Effects of FC on the histological characteristics of NC/Nga mice with HDM-induced AD. (**A**) Scheme of the experimental schedule. (**B**) Clinical features of AD-skin symptoms on day 42. (**C**) Dermatitis scores were measured once a week for 6 weeks. The dermatitis score was the average value of the sum of scores for each symptom. (**D**) Trans-epidermal water loss (TEWL) was measured at end of 6 weeks. (**E**) Dorsal protein level of IL-6 were quantified by ELISA kit. The data shown represent mean ± SD (n = 6) of three independent experiments. ^###^p < 0.001 vs the control group; **p < 0.01, and ***p < 0.001 vs HDM-induced AD group.

NC/Nga male mice (20-25 g body weight, 6 weeks old) were obtained from Daehan BioLink (Eumsung, Korea) and they were kept under standard conditions at a temperature of 22–25 °C, humidity of 40–60%, and a 12-h light/dark cycle with free access to food and tap water. After acclimation for 1 week, their dorsal hair was removed using an electric shaver and they were randomly assigned to one of six groups (n = 6 per group, total 36): (1) a baseline-applied normal group (NOR), (2) a house dust mites (HDM)-sensitized group, (3) Dexamethasone (DEX, positive control), and (4) FC oral administration group (FCO), (5) FC topical administration group (FCT), and (6) FC oral + topical administration group (FCOT). To induce AD-like skin lesions, the shaved dorsal area was topically treated with of 100 mg crude extract of HDM (AD cream, Biostir-AD; Biostir, Hyogo, Japan) twice a week for 6 weeks. For the disruption of the skin barrier, the dorsal skin of mice was applied by 150 μL of 4% sodium dodecyl sulfate (SDS), before 3 h of HDM application. The normal group was treated with the same volume of baseline as vehicle. At 14 days of first AD induction, the mice were topically applied vehicle, administrated orally with DEX (5 mg/kg dissolved in PBS), or administered FC (100 mg/kg) according to the conditions of each group 4 h after HMD treatment. In the FCOT group, half of the concentration was applied orally and topically. The experimental scheme is summarized in Fig. 1A.

### Animal sacrifice

On the 43th day, experimental mice were sacrificed after the measurement of body weight, dorsal dermatitis score, and TEWL. Each mouse was placed in the visible chamber and anesthetized with CO_2_ gas. Then, blood samples were obtained through heart puncture into heparinized tubes and cervical dislocation was performed. The serum was collected by centrifugation at 2000*g* for 20 min at 4 °C. Truncated dorsal skin tissues were isolated for subsequent experiments. These samples were stored at –80 °C until further use. The study is reported in accordance with ARRIVE guidelines (https://arriveguidelines.org).

### Measurement of dermatitis score

The dermatitis severity evaluation of HDM-induced AD in mice was checked once a week, according to the scoring system as followed. Including edema, scarring/dryness, erythema/hemorrhage, and excoriation/erosion as major symptoms, the dorsal dermatitis scoring was, respectively, scored as 0 (none), 1 (mild; < 20%), 2 (moderate; 20–60%) and 3 (severe; > 60%), and lastly described as the sum of the individual scores.

### Trans-epidermal water loss (TEWL)

To investigate the effect on dorsal skin dryness of NC/Nga mice, we assessed the value of TEWL using gpskin barrier light (GPower, Seoul, Republic of Korea). On the 43th day, TEWL was measured at the center of the shaved dorsum area of mouse under specific conditions at approximately 21 ± 2 °C and 50–55% humidity. The data were measured once the TEWL readings reached a state of equilibrium, approximately 30 s post the probe's placement on the skin. The statistical value was shown in terms of fold change by comparison with the control group and subsequently represented in units of g/m^2^/h.

### Histological analysis

At the dissection day, the dorsal skin was fixed with 10% formalin, embedded in paraffin, and sectioned into 4 μM slices. Furthermore, the selected sections were stained with hematoxylin and eosin (H&E) and toluidine blue for evaluation of pathological changes and the infiltration of mast cell, respectively. Images were captured under an optical microscope (Leica, Wetzlar, Germany) using the Leica software. Through these two staining analysis, each group was measured at least 30 times. In immunohistochemical (IHC) staining, the slides were deparaffinized and then blocked using 0.6% H_2_O_2_ in 50% MeOH for endogenous peroxidase activity. These slides were treated with 0.3% Triton in phosphate-buffered solution and pre-blocking with 10% normal goat serum for 1 h. The sections were incubated with a specific antibody for overnight at 4 °C. Next day, they were washed and incubated with horseradish peroxidase-conjugated secondary antibodies for 1 h. The activated slides were treated with 3, 3-diaminobenzidine chromogen and counterstained with H&E. Histological changes in all the stained skin sections were observed using a DM IL LED microscope (Leica Microsystems, Wetzlar, Germany) and photographed using a DFC295 camera (Leica Microsystems). Digital images were captured from each slide and analyzed using Leica Application Suite (Leica Microsystems).

### Cytokine analysis

Dorsal protein samples were extracted by suspending with PRO-PREP™ protein extraction solution (Intron Biotechnology Inc., Seoul, Korea) and centrifuging the debris at 11,000×*g* for 30 min at 4 °C. After rapid freezing of their supernatant for storage, the protein concentration was determined using the Bio-Rad protein assay reagent (Bio-Rad Laboratories Inc., Hercules, CA, USA) according to the manufacturer’s protocol. The level of IL-6 was quantified using an ELISA kit according to the manufacturer’s protocol.

### RT-qPCR analysis

Total RNA was extracted from dorsal skin tissues using an RNeasy Fibrous Tissue Mini Kit (Qiagen, Valencia, CA, USA) according to the manufacturer’s protocol. cDNA was obtained using isolated total RNA (2 μg), d(T)16 primer, and AMV reverse transcriptase. Relative gene expression was measured by quantitative reverse transcription polymerase chain reaction (RT-PCR; 7500 Real-Time PCR System, Applied Biosystems, Foster City, CA, USA) with SYBR premix Ex Taq. The data was calculated based on the cycle threshold (Ct) values using the ΔΔCt method of quantification. The results were expressed as the optical density ratio relative to GAPDH. The sequences of real-time reverse transcription polymerase chain reaction (RT-PCR) primers used were as follows;

mouse IL-13, forward 5′‑AACGGCAGCATGGTATGGAGTG-3′, and reverse 5′‑TGGGTCCTGTAGATGGCATTGC-3′; mouse TNF-α, forward 5′‑ATGAGCACAGAAAGCATGAT-3′, and reverse 5′‑TACAGGCTTGTCACTCGAAT-3′; and mouse GAPDH, forward 5′‑GACGGCCGCATCTTCTTGT-3′, and reverse 5′‑CACACCGACCTTCACCATTTT-3′.

### Western blot analysis

Protein extracts from the dorsal skin and HaCaT keratinocytes were prepared using PRO-PREP™ protein extraction solution and homogenized at 4 °C. As previously stated, tissue or cell debris of the supernatant was removed with micro-centrifugation followed by immediate freezing and then the protein concentration was determined using the Bio-Rad protein assay reagent, according to the manufacturer’s protocol. After the electrophoresis of 8–12% SDS–polyacrylamide gel, proteins from each group were electroblotted on a polyvinylidene difluoride (PVDF) membrane. The immunoblot was incubated with a blocking solution (2.5–5% skim milk) for 30 min at room temperature and incubated overnight with a primary antibody (dilution, 1:1000 in Tween 20/Tris-buffered saline (T/TBS)) at 4 °C. After washing thrice with T/TBS, the blots were incubated with a horseradish peroxidase-conjugated secondary antibody (dilution, 1:2000) for 2 h at room temperature and washed again thrice with T/TBS. The blots visualized using an enhanced chemiluminescence system (GE Healthcare, WI, USA). Densitometry was performed using Bio-Rad Quantity One software (version 4.3.0; Bio-Rad Laboratories, Inc.).

### Cell culture and sample treatment

The human keratinocyte HaCaT cell line were purchased from CLS Cell Lines Service (Eppelheim, Baden-Württemberg, Germany), and were grown at 37 °C in DMEM supplemented with 10% (v/v) FBS and antibiotics (100 U·mL −1 penicillin and 100 μg·mL −1 streptomycin) at 37 °C in a humidified 5% CO_2_ incubator. HaCaT keratinocytes were seeded at a density of 1 × 10^5^ cells/mL per well and incubated for 24 h. The medium was changed to serum free medium with FC (125, 250, or 500 μg/mL) and incubated 1 h. The cells were stimulated with a TNF-α and interferon gamma (IFN)-γ mixture (10 ng/mL, each) for the indicated time.

### Cell viability assay

The viability of HaCaT keratinocytes was assessed using the colorimetric MTT assay. Cells were seeded in 96-well culture plate at 5 × 10^4^ cells/mL in culture medium and allowed to attach for 24 h. Next, the cells were treated with medium containing various concentrations of FC and incubated for 24 h. The following day, the cells were kept in 50 μL of MTT (5 mg/mL) for 4 h at 37 °C and DMSO was used to dissolve the formazan precipitate after removal of the supernatant. Cell viability was measured at 540 nm using an Epoch microplate spectrometer (BioTek, Winooski, VT, USA).

### Statistical analysis

Data were expressed as mean ± SD of triplicate experiments. Comparisons among groups were carried out using one-way analysis of variance followed by Dunnett’s post-hoc test using GraphPad Prism 5 (GraphPad Software, San Diego, CA, USA). p values < 0.05 were considered statistically significant.

### Ethics approval and consent to participate

All animal procedures were performed in accordance with the Guidelines for the Care and Use of Laboratory Animals and approved by the Institutional Animal Care and Use Committee (IACUC) of Sangji University (approval protocol no. 2020-9). The study is reported in accordance with ARRIVE guidelines (https://arriveguidelines.org).

## Results

### FC attenuates AD-like clinical symptoms of HDM-induced AD skin in NC/Nga mice

As the results of the experiment based on the schedule shown in Fig. 1A, continuous application of HDM worsened the visual symptoms and dermatitis score of AD, such as edema, erythema, erosion, dryness and excoriation. By contrast, regardless of the FC administration methods, FC treatment downregulated AD symptoms (Fig. 1B,C). TEWL value, as the representative of skin hydration and barrier state, was evaluated in the dorsum on the last day of 6 weeks. All FC administrations mitigated TEWL value in contrast to increased expression of HDM treated group. The specific average and standard deviation (SD) values of TEWL (g/m^2^/h) for each group are as follows: NOR 3.600 ± 1.516, HDM 11.750 ± 2.061, DEX 4.000 ± 1.870, FCO 5.000 ± 1.264, FCT 4.333 ± 2.581, and FCOT 5.667 ± 2.875 (all values with p < 0.001) (Fig. 1D). Further, IL-6 production, the primary pro-inflammatory maker, was alleviated by FC administration. In FCO and FCT groups, the reduction degree was similar to that of the positive control group, and the FCOT group had the finest reduction effect (Fig. 1E).

### FC inhibits epidermal hyperplasia and mast cell infiltration of HDM-induced AD skin in NC/Nga mice

**Figure 2:**
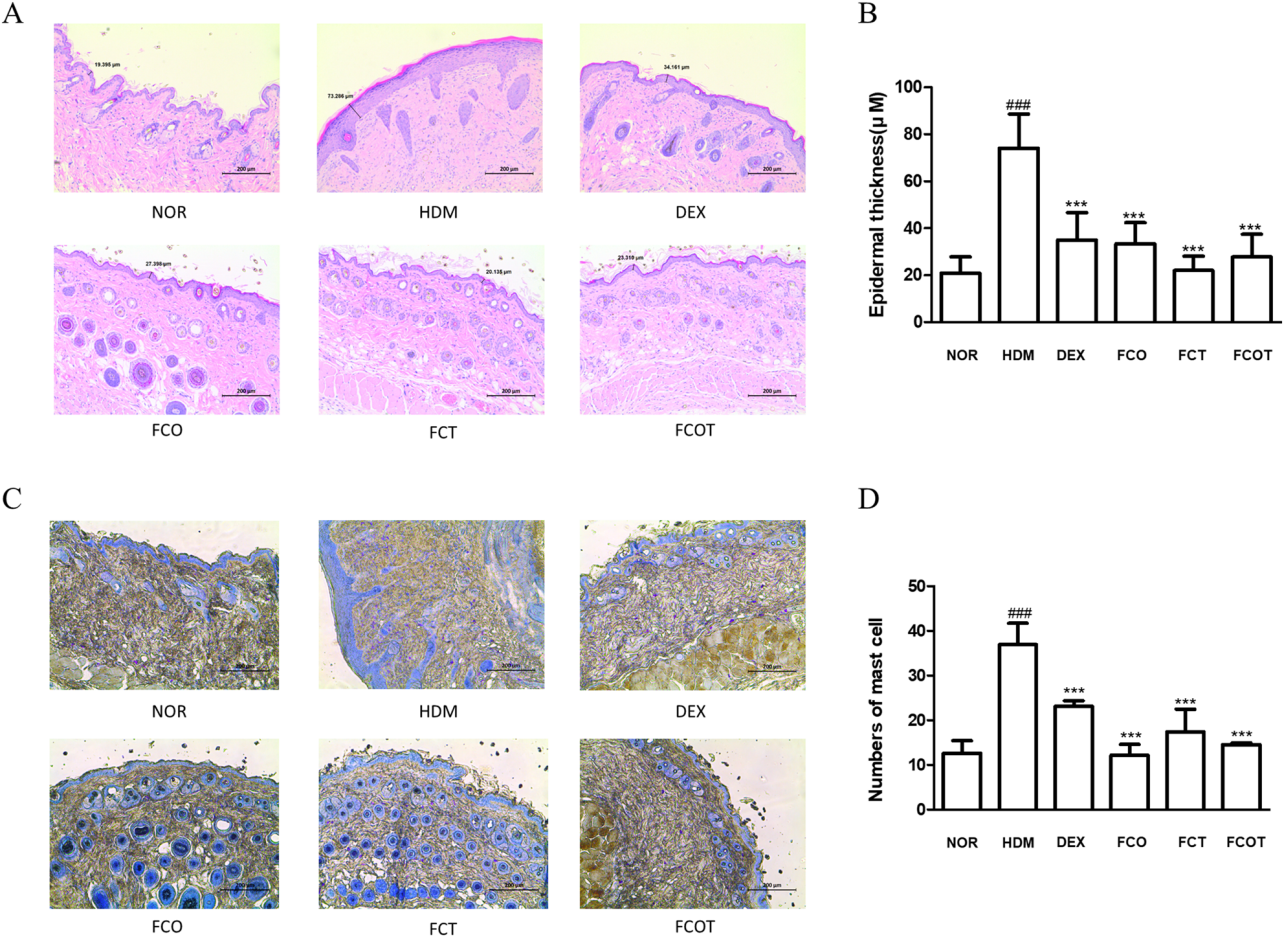
Effect of FC on epidermal thickness and mast cell infiltration of NC/Nga mice with HDM-induced AD. (**A**,**B**) Epidermal thickness was examined by H&E staining of skin lesions (scale bar = 200 μm). (**C**,**D**) Mast cell infiltration in mouse skin lesions was measured by toluidine blue staining (scale bar = 200 μm). Measurement of epidermal thickness and mast cell in stained sections were measured under a microscope and expressed as the average total count in five fields. The data shown represent mean ± SD (n = 6) of three independent experiments. ^###^p < 0.001 vs the control group; ***p < 0.001 vs HDM-induced AD group.

To investigate the anti-inflammatory effect of FC in cutaneous tissue, epidermal thickness, and mast cell infiltration were estimated by histological examination. Through the H&E staining, epidermal hyperplasia and hyperkeratosis were shown in the HDM-induced AD group. Contrariwise, FC treatment attenuated thickened epidermis and its reduction effect was similar to or better than that of the positive control group in all methods DEX. The specific average and SD values of epidermal thickness (μm) for each group are as follows: NOR 20.911 ± 6.861, HDM 74.122 ± 14.544, DEX 35.051 ± 11.606, FCO 33.436 ± 8.963, FCT 22.020 ± 6.081, and FCOT 27.810 ± 9.616 (all values with p < 0.001) (Fig. 2A,B). As shown in Fig. 2C and D, repeated treatment with HDM significantly increased the number of mast cell infiltration, whereas treatment with DEX and FC significantly decreased them. The reduction effect of FC was the best in the oral-treated group. The specific average and SD values of the numbers of mast cells for each group are as follows: NOR 12.657 ± 2.794, HDM 36.952 ± 4.723, DEX 23.133 ± 1.225, FCO 12.185 ± 2.437, FCT 17.417 ± 5.067, and FCOT 14.546 ± 0.418 (all values with p < 0.001). In that the skin hyperplasia and immune cells observed above are primary hallmarks of AD concerning skin inflammation, all FC administration methods showed potential for relieving skin inflammation.

### FC suppresses the mRNA expression of pro-inflammatory cytokines in HDM-induced AD mice

**Figure 3:**
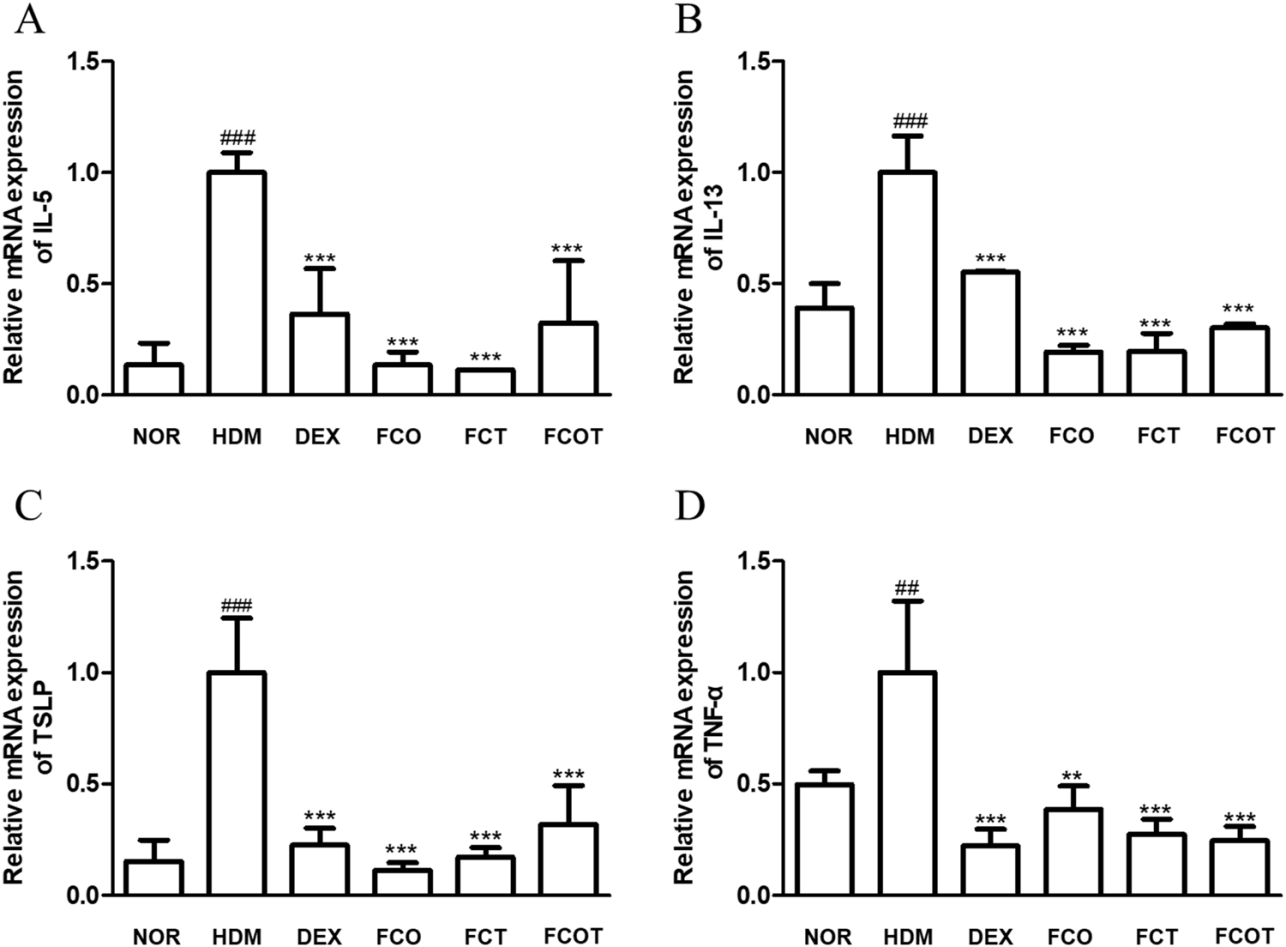
Effects of FC on the expression of AD-related chemokine and cytokines of NC/Nga mice with HDM-induced AD. Total RNA prepared from the dorsum tissue, and the expression levels of (**A**) IL-5, (**B**) IL-13, (**C**) TSLP and (**D**) TNF-a were determined by RT-qPCR. The data shown represent mean ± SD (n = 6) of three independent experiments. ^##^p < 0.01, ^###^p < 0.001 vs the control group; **p < 0.01, and ***p < 0.001 vs HDM-induced AD group.

To figure out whether FC inhibits the mRNA expression levels of AD-related cytokines, we measured IL-5, IL-13, TSLP, and TNF-α by using qRT-PCR. These major cytokines are known as pro-inflammatory and allergy-mediating cytokines in the pathogenesis of skin disorders^15^. HDM treatment increased the mRNA levels of all cytokines, but all FC-administered groups decreased them with significance (Fig. 3).

### FC regulates the expression of AD-related cytokines in HDM-induced AD mice

**Figure 4:**
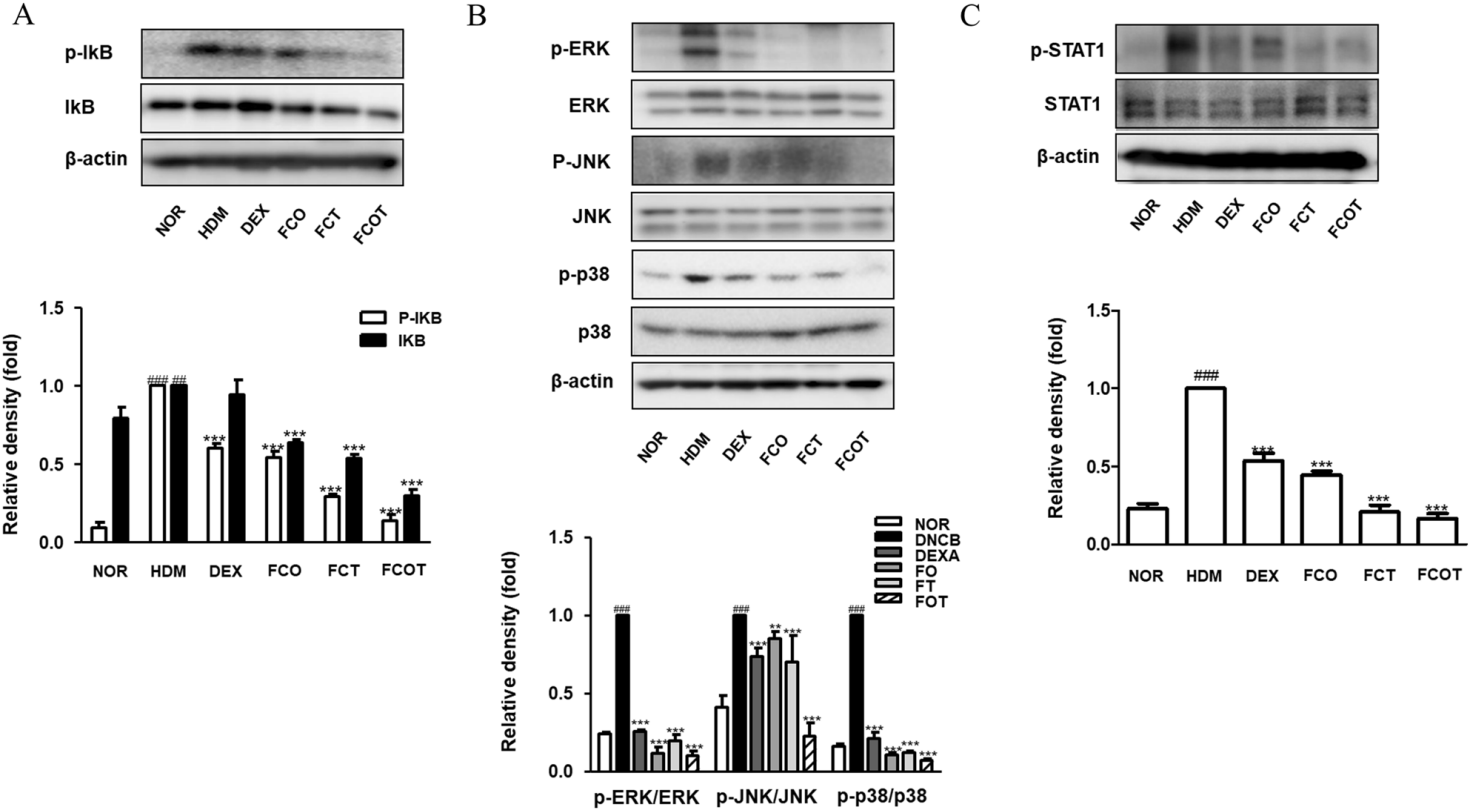
Effects of FC on the phosphorylation of IκBα, MAPKs, and STAT1 in NC/Nga mice with HDM-induced AD. Total proteins were prepared and immunoblotted for (**A**) p-IκB, IκB, (**B**) p-ERK, ERK, p-JNK, JNK, p-p38, p38, (**C**) p-STAT1, and STAT1 by using specific antibodies. β-actin was used as internal control. The protein levels were quantified by band density in proportion to the expression of total form. The original result images of all western blots are attached in the Supplementary Figures. The data shown represent mean ± SD of three independent experiments. ^##^p < 0.01, ^###^p < 0.001 vs the control group; **p < 0.01, and ***p < 0.001 vs HDM-induced AD group.

As prominent inflammatory cytokine regulators, NF-κB and MAPK pathways are known to play important roles by influencing each other^16^. As one of the representative factors of NF-κB pathway, the phosphorylation of IκB was increased in HDM-induced AD mice dorsal protein, however, all FC treatments decreased them (Fig. 4A). With respect to MAPKs pathway, MAPK families in the HDM group, significantly increased expression levels of ERK, JNK, and p38 were reduced by FC administration. The degree of phosphorylation inhibition of JNK was most evident in the combined treated group, and the degree of phosphorylation inhibition of ERK and p38 was similar in all FC treated groups (Fig. 4B). Based on in vitro results, the STAT1 phosphorylation inhibitory effect was confirmed. The increased phosphorylation expression by HDM treatment was inhibited by FC treatment, and the degree was most pronounced in FCT and FCOT groups (Fig. 4C).

### FC has not cytotoxic effect in HaCaT keratinocytes and regulates the expression of atopy mechanistic proteins in TNF-α/IFN-γ-stimulated HaCaT keratinocytes

**Figure 5:**
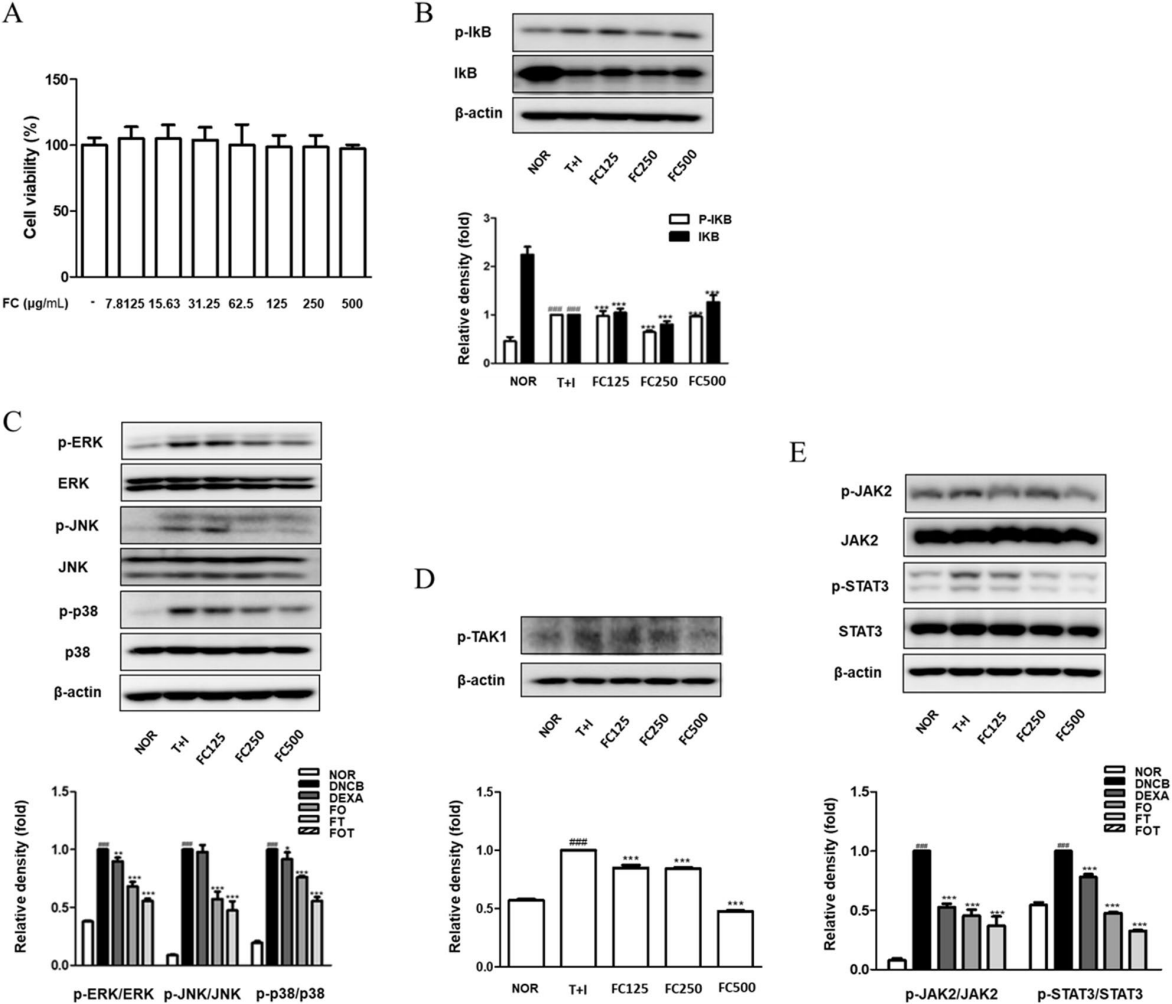
Cytotoxic effect of FC on HaCaT keratinocytes and effects of FC on the phosphorylation of IκBα, MAPKs, and JAK2/STAT3 in TNF-α/IFN-γ-stimulated HaCaT keratinocytes. (**A**) Cells were treated with various concentrations of FC for 24 h, and cell viability was measured by MTT assay. *p < 0.05, **p < 0.01, and ***p < 0.001 vs FC non-treated group. Total proteins were prepared and immunoblotted for (**B**) p-IkB, IkB, (**C**) p-ERK, ERK, p-JNK, JNK, p-p38, p38, (**D**) p-TAK1, (**E**) p-JAK2, JAK2, p-STAT3, and STAT3 by using specific antibodies. β-Actin was used as internal control. The protein levels were quantified by band density in proportion to the expression of total form. The original result images of all western blots are attached in the Supplementary Figures. The data shown represent mean ± SD of three independent experiments. ^###^p < 0.001 vs the control group; *p < 0.05, **p < 0.01, and ***p < 0.001 vs TNF-α/IFN-γ-stimulated group.

Above all, the effect of FC on cell viability was examined using the MTT assay. All concentrations of FC treatment at 7.8125–500 μg/mL did not show cytotoxicity in HaCaT keratinocytes (Fig. 5A). Therefore, further experiments were performed at concentrations of 100, 250, and 500 μg/mL. In agreement with the results of in vivo model, we conducted western blot assay to examine whether FC could suppress the expression of TNF-α/IFN-γ-induced skin-inflammatory pathways. As a result, the treatment of FC on the TNF-α/IFN-γ-induced signal cascade of NF-κB activation inhibited IκBα phosphorylation and degradation in vitro (Fig. 5B). As the regulatory effect of FC on TNF-α/IFN-γ-induced MAPKs pathway, FC treatments reduced each activity of ERK, JNK, and p38 (Fig. 5C). The MAPKs pathway is associated with NF-κB activity, and both mechanisms are involved in TAK cascade as a common upstream factor^17^. The activation of TAK was reduced by FC treatment in contrast to that of to the TNF-α/IFN-γ-treated group (Fig. 5D). These results tallied with the suggestion that FC hinders the activation of IκB and MAPKs pathways in TNF-α/IFN-γ-induced HaCaT Keratinocytes. Furthermore, STAT signaling was detected as the one of the critical signaling pathways in skin inflammation^18^. All FC treatment concentrations significantly inhibited the phosphorylation of STAT3 and its upstream JAK2 in TNF-α/IFN-γ-stimulated groups (Fig. 5E). Thus, based on all of the above results, the down-regulatory effects of FC on skin inflammatory pathways may be presented as an AD-therapeutic strategy in addition to the previously reported skin effectiveness.

### FC restores the expression of filaggrin, skin barrier proteins, in vivo and in vitro

**Figure 6:**
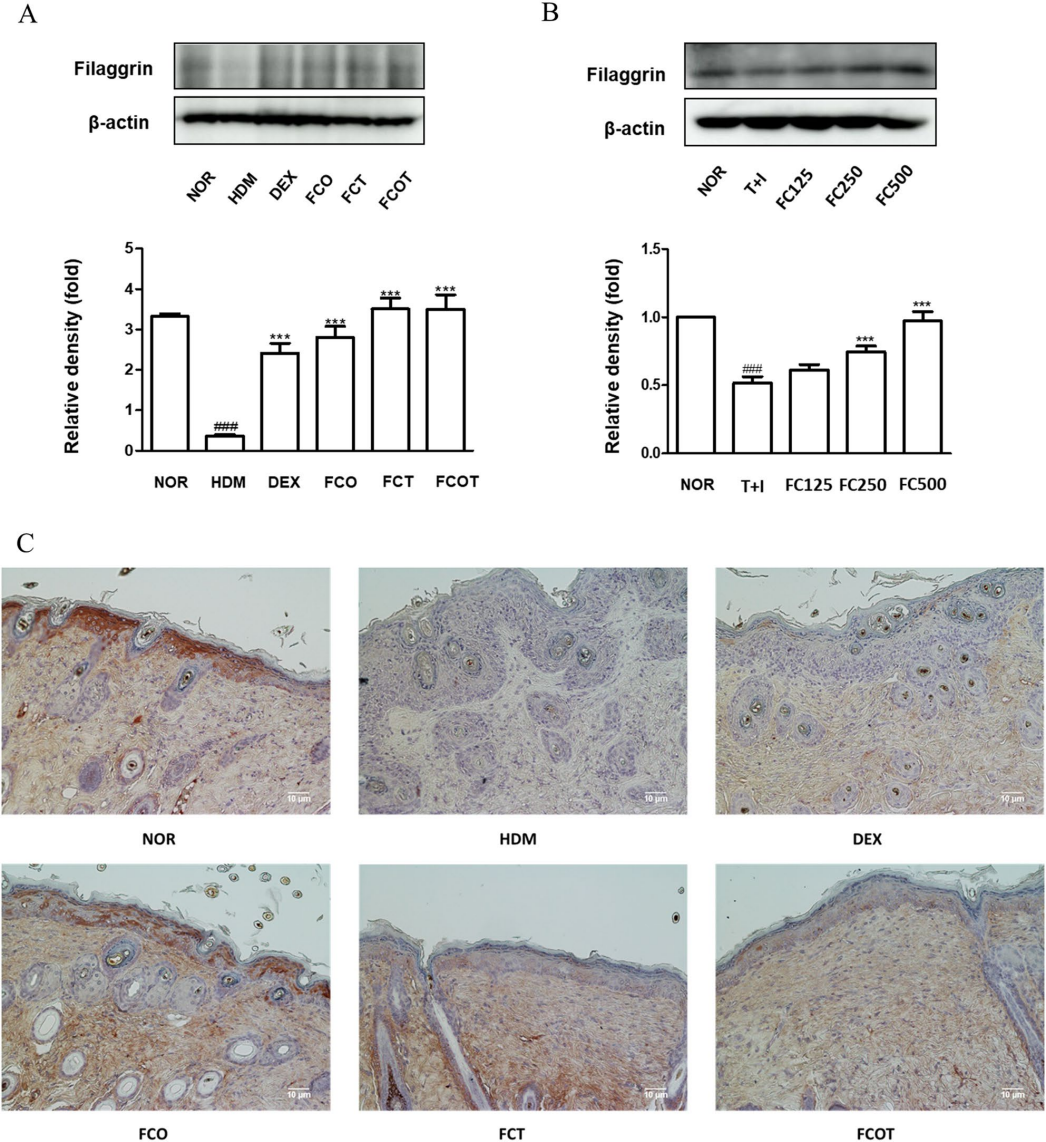
Effects of FC on the expression of Filaggrin in vivo and in vitro models. (**A**,**B**) Total proteins were prepared and immunoblotted for Filaggrin by using specific antibodies. β-Actin was used as internal control. The protein levels were quantified by band density in proportion to the expression of total form. The original result images of all western blots are attached in the Supplementary Figures. (**C**) Representative histologic section findings of skin dorsal tissue immunostained with Filaggrin antibody using immunohistochemical staining (scale bar = 10 μm). The data shown represent mean ±  SD of three independent experiments. ^###^p < 0.001 vs the control group; ***p < 0.001 vs TNF-α/IFN-γ-stimulated group.

Filaggrin plays a main epidermal barrier by maintaining the structural coherence of the stratum corneum^19^. We investigated the variations in the amount of filaggrin through WB and IHC analysis. In vivo model, the decreased filaggrin expression in the HDM treated group compared to the normal group was recovered by DEX treatment and FC treatment. All except the combined treatment with FC were significant (Fig. 6A). As shown in Figs. 6C, IHC analysis of filaggrin showed distinct brown expression in epidermis of the normal group and increased brown expression in the DEX- and all FC-treated groups. Conversely, the filaggrin expression was almost absent in the HDM treated group. In vitro model, the filaggrin expression was noticeably reduced in TNF-α/IFN-γ-stimulated groups, whereas expression was significantly improved in the medium and high concentration FC groups (Fig. 6B).

## Discussion

AD is a representative inflammatory disease developing locally and systemically in the skin, which has obscured causes and treatments^20^. Since existing therapies impose a financial burden and side effects on AD patients, research on the development of novel therapies has been conducted for a long time^7^. In this study, the anti-atopic efficacy of FC was demonstrated in HaCaT keratinocytes and HDM-sensitized NC/Nga mice model.

HaCaT cell line is an epithelial keratinocyte as one of the representative keratinocytes used in AD experiments. In AD patients, keratinocytes are activated by pervading antigens in unsound skin barrier and then, aggravate a skewed Th2 immune response, which further degenerate immune disproportion and itching^21,22^. As in vivo model, NC/Nga mouse is a generally used mouse strain for the research of AD mechanisms, especially inbreeding to arouse AD symptoms, such as eczema and pruritus^20^. This murine strain need to irritate their skin by reapplied allergens to totally develop AD, so we applicate chemical substance SDS and HDM allergen. Among HDM allergens, we applied *Dermatophagoides farinae* to this experiment as an AD inducer, the most commonly used aeroallergens^23,24^. This allergen is known to cause various allergic inflammatory diseases by triggering pro-inflammatory mediators via MAPKs, IκB, or STATs pathways^24^.

As a main extracellular matrix structural protein, collagen serves as a connector for skin and bone of animal organisms^25^. Therefore, collagen and its peptides have been revealed to have inhibitory activities against inflammation, bacteria, fungi, and immunity, so it could be a classic example of 'cosmeceuticals' in the emerging field of medical cosmetics^26^. In the past, collagen extracted from mammals was usually used in cosmeceuticals, but recently, collagen obtained from marine origin has been in the spotlight due to religious issues, potential risks related to animal diseases, and sustainable socioeconomic and environmental issues^27,28^. Although FC has been shown to have anti-aging, anti-inflammatory, and wound-healing effects^29^, the AD mitigating effects of FC and which route of administration are most effective have not been investigated.

The pathogenesis of AD is multifactorial and interactive, deeply involving immune abnormality and epidermal barrier disruption^30^. Stimulated immune Th cells produce diverse cytokines and chemokines, which modify the epidermal differentiation and decrease the skin barrier related proteins, like filaggrin^31^. From the perspective of inflammatory cascade in AD, activated NF-κB and amplified pro-inflammatory mediators tend to exacerbate immune response by interacting with each other^30^. The NF-κB dimer is divided into phosphorylated and ubiquitinated IκB-α and then, transported into the nuclei for the differentiation of T cells and transcription of pro-inflammatory cytokines^4^. As follows; differentiated Th2 cells secrete IL-4, IL-5, IL-6, and IL-13 cytokines^2,30^. TNF-α and IL-6 encourage the phosphorylation of MAPKs signaling factors, inducing cytokines activation as the upstream pathways of NF-κB. STAT protein family is also transcription factors transmitting inflammatory mediators by binding to DNA^4,32^. In AD condition, TSLP secreted from keratinocytes can transform naive T cells into Th2 cells by stimulating several immune cells, directly or indirectly. Furthermore, TSLP is known to be relevant to cutaneous pruritus by interacting with STATs pathways^33,34^. From the skin barrier point, the firmly maintained skin barrier collapses due to the influx of outside irritants and inflammatory response, so cross-linked barrier proteins are disrupted and gradually lose epidermal integrity with their protective function^35^. At the outermost of the epidermal barrier, filaggrin protein acts as a defense component by converting free amino acids into natural moisturizing factors^2^. The molecular mechanisms mentioned above are known to be involved, but particularly the STAT3 signaling pathway downregulates filaggrin expression^36^.

In turn, immune response and skin barrier disruption mutually exacerbate the vicious loop of AD via these various signaling mechanisms^37^. By clarifying the molecular mechanisms of FC in vitro and in vivo (Figs. 4 and 5), FC repressed the stimulation of IκB, MAPKs, STATs pathways, and their upstream pathways, which are typical intracellular pathways involved in inflammation and skin barrier dysfunction. These inflammatory pathways are known to be regulated according to the synthesis of pro-inflammatory mediators confirmed that FC treatment reduced their activation in this study as well (Figs. 1E and 3). The expression of filaggrin was also restored in vivo and in vitro model (Fig. 6). At present experiment, FC attenuated visible phenotype and histological phenomena of AD, including skin irritation, lichenization, bleeding scab, epidermal hyperplasia, and increased mast cell infiltration (Figs. 1B,C, and 2). Despite slight differences in the expression depending on the administration routes, the application of FC showed a significant effect of relieving skin inflammation and restoring skin barrier in AD regardless of the administration method. To elaborate further, when an equal amount of FC was administered, it was observed that applying it through the combination of both oral administration and skin application yielded better results in terms of mechanistic pathways and enhancing skin barrier integrity, compared to a single method of administration.

For all confirming the effectiveness of FC through this and previous experiments^38–41^, there may be concerns about limiting the permeability within the skin when applied to the dorsal skin due to the molecular size of FC. Because of its molecular weight, the mechanism of topically applied FC involves direct interactions with the skin's surface and underlying layers. When applied to the skin, FC remains emulsified on the epidermal layer, forming a thin protective barrier that prevents moisture evaporation and safeguards the skin from external stimuli. Besides, some research papers confirmed different skin application effects based on molecular size. When applying the same sample topically to the skin, the application of high molecular weight increased the stratum corneum's moisture content, while the application of low molecular weight tended to enhance the fluidity of the lipid layer^42–44^. Moreover, to address these limitations and enhance effective absorption, current research is actively exploring various formulation methods for FC application, such as the formation of films or the use of highly permeable materials like sticker patches^36,45–50^. In this regard, this study on FC's effects on AD holds significant value and there is still a lack of extensive pharmacokinetic research in this area, indicating a need for further investigations.

**Figure 7:**
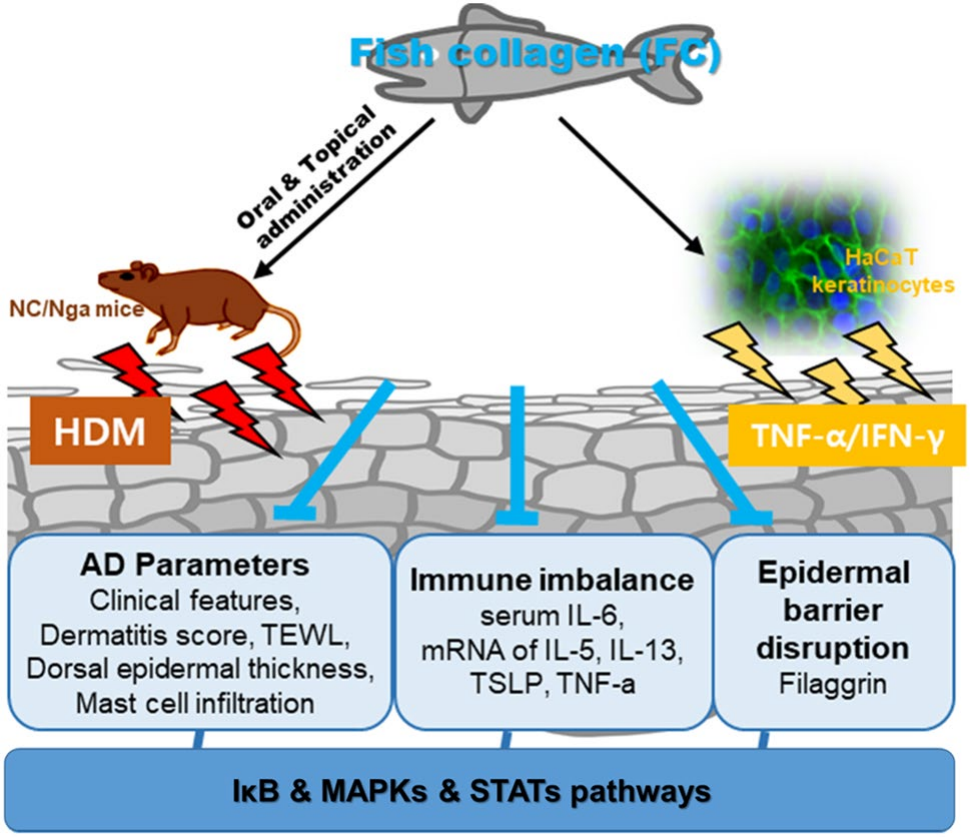
A graphical abstract for illustrating the role of FC on House Dust Mite-induced mice and HaCaT keratinocytes. In vivo and in vitro models, FC alleviated AD symptoms by regulating immune response and skin barrier dysfunction are both mitigated via the regulation of IκB, MAPKs, and STATs pathways.

Through in vivo and in vitro experiments, we found that administration of FC alleviated the expression of pro-inflammatory cytokines and mast cell infiltration in terms of anti-allergic properties, and restored the level of filaggrin, epidermal hyperplasia, and TEWL in terms of skin barrier function via the regulation of IκB, MAPKs, STATs pathways. We augmented the anti-AD efficacy of FC as a representative cosmeceuticals and further suggested the possibility of commercialization of FC as an adjuvant and treatment for AD through deeper mechanism research and combination studies for high penetration and bioabsorbability (Fig. 7).

### Supplementary Information

Supplementary Figures.

## Data Availability

The datasets generated and/or analyzed during the current study are available from the corresponding author on reasonable request.
