# Supplementary material for: Anti-atopic dermatitis effect of fish collagen on house dust mite-induced mice and HaCaT keratinocytes
Source: Sci Rep. 2023 Sep 9;13:14888. doi: 10.1038/s41598-023-41831-w (PMC10492863; doi:10.1038/s41598-023-41831-w)

Figure 4A p- IκB


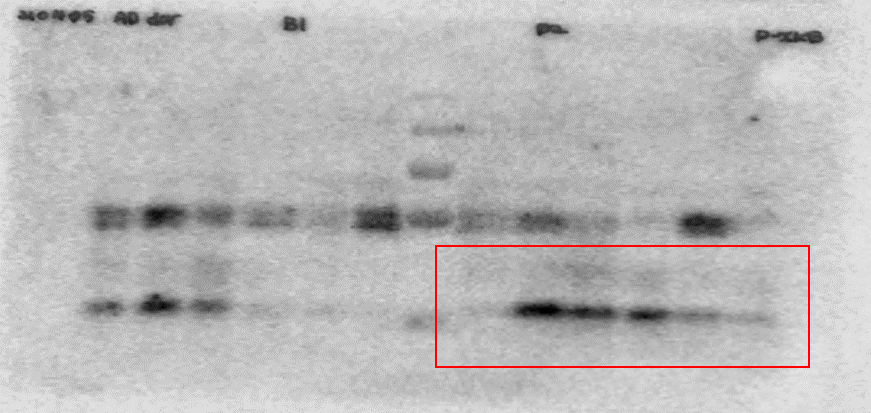


Figure 4A IκB


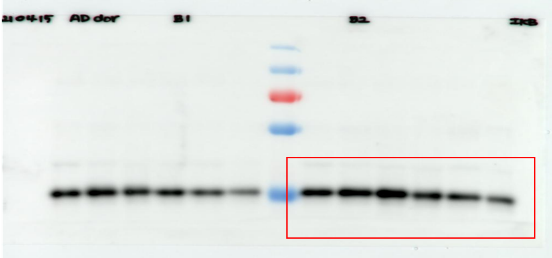


Figure 4A 𝛽-actin


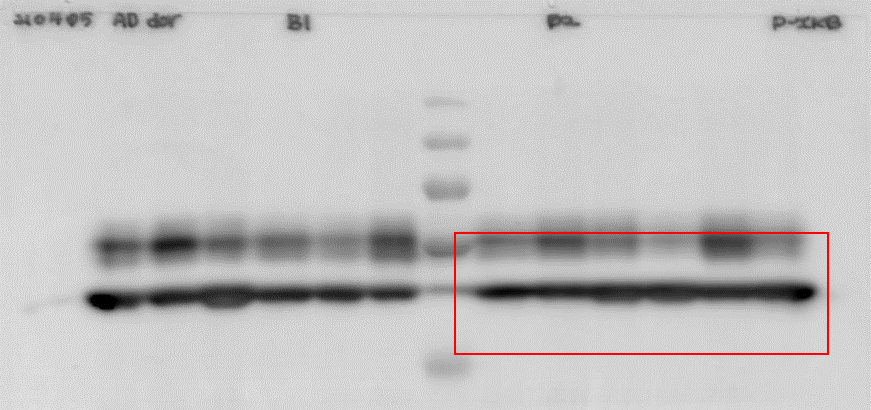


Figure 4B p-ERK


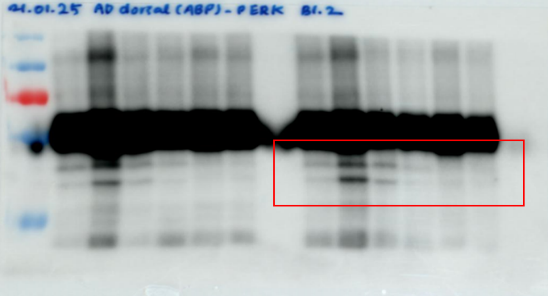


Figure 4B ERK


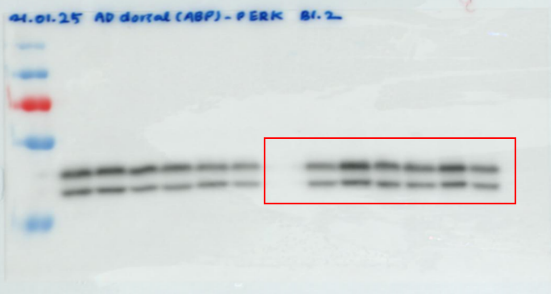


Figure 4B p-JNK


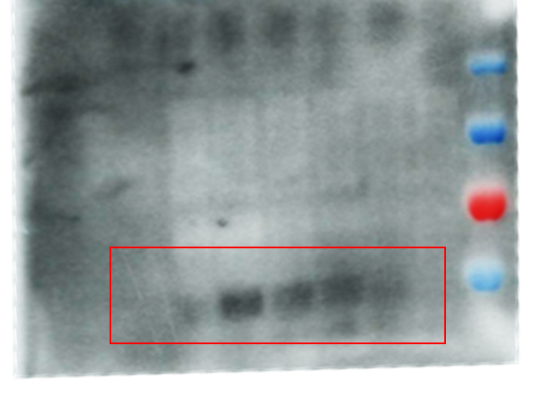


Figure 4B JNK


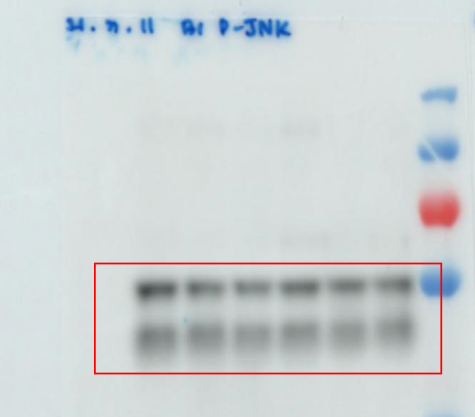


Figure 4B 𝛽-actin


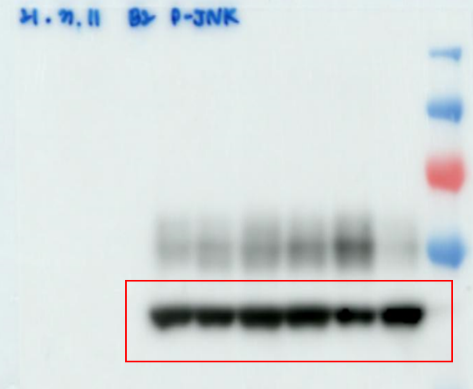


Figure 4B p-p38


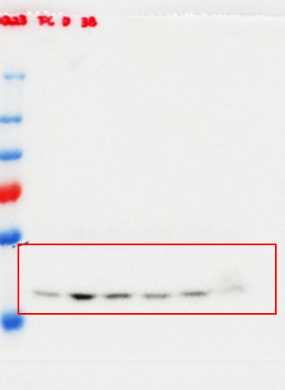


Figure 4B p38


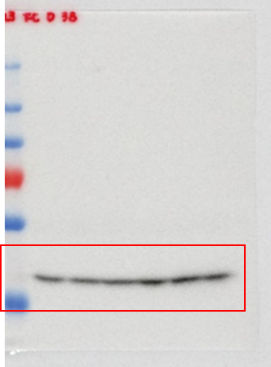


Figure 4B 𝛽-actin


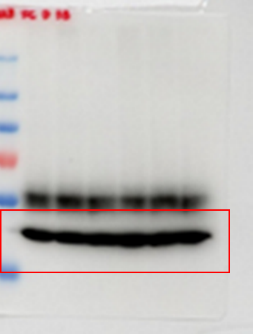


Figure 4C p-STAT1


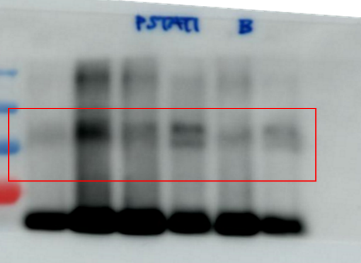


Figure 4C STAT1


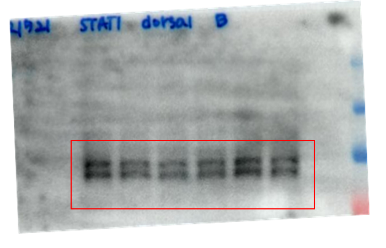


Figure 4C 𝛽-actin


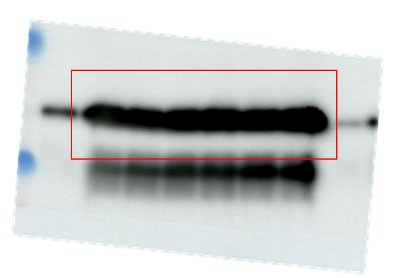


Figure 5B p- IκB


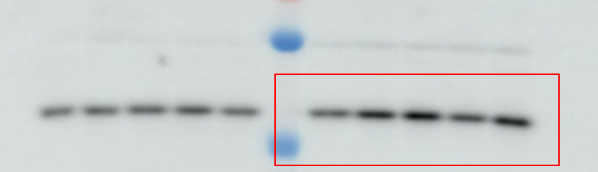


Figure 5B IκB


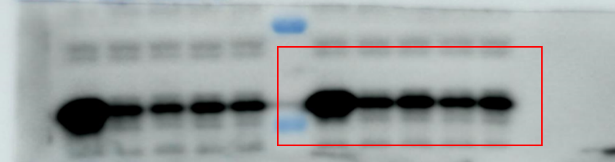


Figure 5B 𝛽-actin


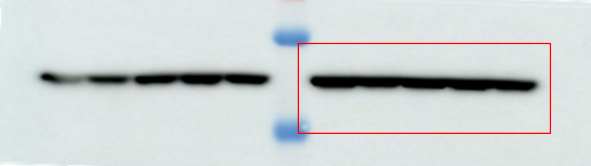


Figure 5C p-ERK


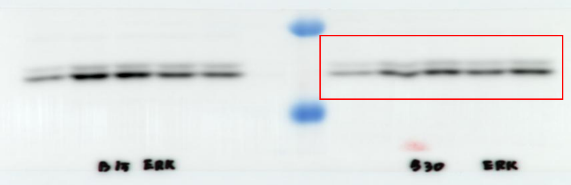


Figure 5C ERK


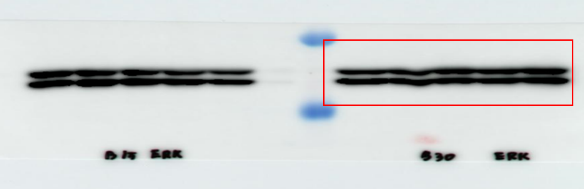


Figure 5C 𝛽-actin


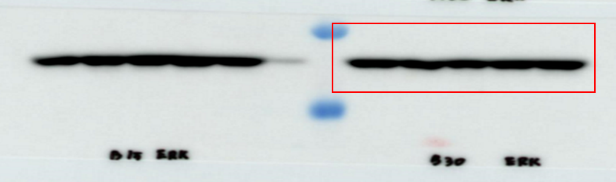


Figure 5C p-JNK


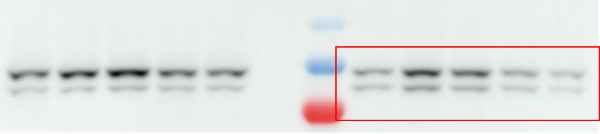


Figure 5C JNK


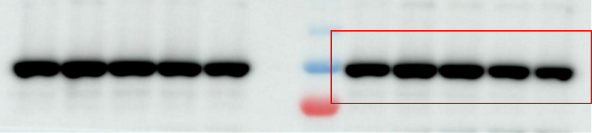


Figure 5C 𝛽-actin


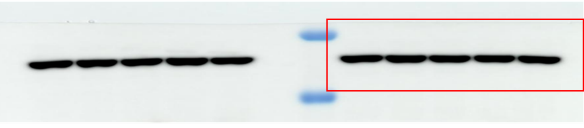


Figure 5C p-p38


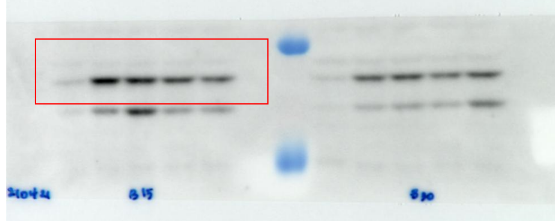


Figure 5C p38


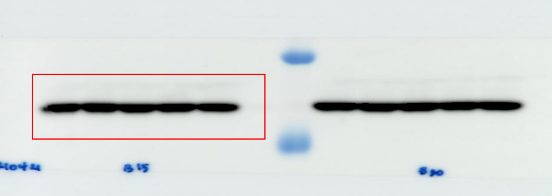


Figure 5C 𝛽-actin


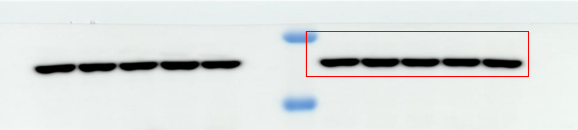


Figure 6A Filaggrin


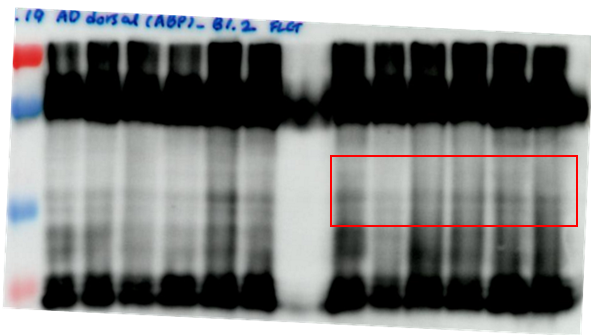


Figure 6A 𝛽-actin


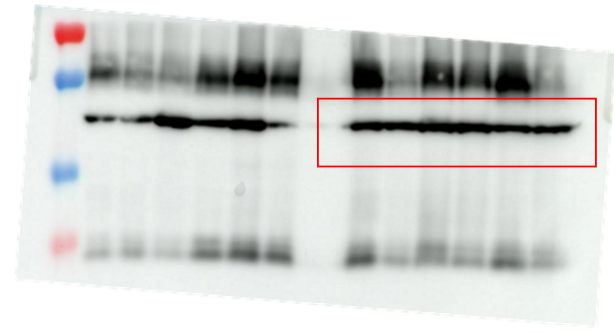


Figure 6B Filaggrin


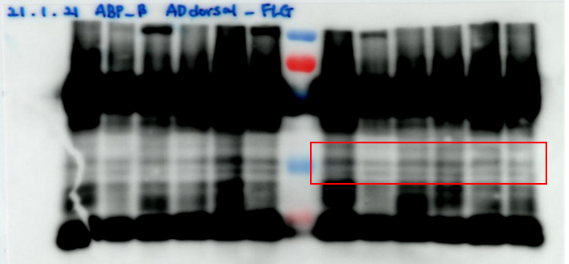


Figure 6B 𝛽-actin


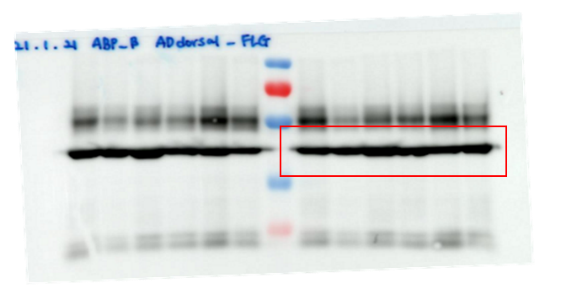

Supplement: Supplementary file 1 — Supplementary Figures. [file 41598_2023_41831_MOESM1_ESM.docx]
